# A Dual Neural Architecture Combined SqueezeNet with OctConv for LiDAR Data Classification

**DOI:** 10.3390/s19224927

**Published:** 2019-11-12

**Authors:** Aili Wang, Minhui Wang, Kaiyuan Jiang, Mengqing Cao, Yuji Iwahori

**Affiliations:** 1 The Higher Educational Key Laboratory for Measuring & Control Technology and Instrumentations of Heilongjiang, Harbin University of Science and Technology, Harbin 150080, China; minhuijy@163.com (M.W.); jiangkaiyuan@hrbust.edu.cn (K.J.); cmq0113@163.com (M.C.); 2 Department of Computer Science, Chubu University, Aichi 487-8501, Japan; iwahori@isc.chubu.ac.jp

**Keywords:** data classification, light detection and ranging (LiDAR), convolutional neural networks (CNNs), SqueezeNet, octave convolution (OctConv)

## Abstract

Light detection and ranging (LiDAR) is a frequently used technique of data acquisition and it is widely used in diverse practical applications. In recent years, deep convolutional neural networks (CNNs) have shown their effectiveness for LiDAR-derived rasterized digital surface models (LiDAR-DSM) data classification. However, many excellent CNNs have too many parameters due to depth and complexity. Meanwhile, traditional CNNs have spatial redundancy because different convolution kernels scan and store information independently. SqueezeNet replaces a part of 3 × 3 convolution kernels in CNNs with 1 × 1 convolution kernels, decomposes the original one convolution layer into two layers, and encapsulates them into a Fire module. This structure can reduce the parameters of network. Octave Convolution (OctConv) pools some feature maps firstly and stores them separately from the feature maps with the original size. It can reduce spatial redundancy by sharing information between the two groups. In this article, in order to improve the accuracy and efficiency of the network simultaneously, Fire modules of SqueezeNet are used to replace the traditional convolution layers in OctConv to form a new dual neural architecture: OctSqueezeNet. Our experiments, conducted using two well-known LiDAR datasets and several classical state-of-the-art classification methods, revealed that our proposed classification approach based on OctSqueezeNet is able to provide competitive advantages in terms of both classification accuracy and computational amount.

## 1. Introduction

Light detection and ranging (LiDAR) technology is an active remote sensing measurement technology which can obtain ground object information by emitting laser to the target [1]. Compared with optical imaging and infrared remote sensing, LiDAR can obtain high-resolution three-dimensional spatial point clouds, elevation models, and other raster-derived data independent of weather conditions through laser beam [2,3]. The data adopted in this article are LiDAR-derived rasterized digital surface models (LiDAR-DSM) which are obtained by rasterizing the point cloud data acquired by the LiDAR system [4]. There is a lot of research on LiDAR-DSM data classification. Priestnall et al. examined methods for extracting surface features from DSM produced by LiDAR [5]. Song et al. modified the crown shape in spectrum by using DSM [6]. Zhou et al. used minimum description length (MDL) and morphology to recognize the buildings [7]. Zhou combined LiDAR height and intensity data to accurately map urban land cover [8]. 

LiDAR-DSM mainly includes terrain changes and feature heights of the target area and it is suitable for classification tasks that distinguish targets with different heights. Thus, the accurate classification of DSM plays an important role in distinguishing different land cover categories. The classification task of LiDAR-DSM data is usually based on the classification of pixels, that is, the interpretation process of remote sensing images [9]. For example, Lodha et al. used the support vector machine (SVM) algorithm to classify LiDAR data and obtained higher accuracy and convincing visual results [10]. Sasaki et al. adopted a decision tree analysis to investigate the average height of each land class [11]. Naidoo et al. used an automated Random Forest modelling approach to classify eight common savanna tree species [12]. Khodadadzadeh et al. developed a new efficient strategy for fusion and classification of hyperspectral and LiDAR data to integrate multiple types of features [13]. Ghamisi et al. proposed a joint classification method which is based on extinction profiles (EPs) features and convolutional neural network (CNN) to improve the classification accuracy [14]. Then Ghamisi et al. proposed to extract the spatial and background information of DSM data in an unsupervised manner to obtain higher classification precision [15]. Wang et al. combined morphological profiles (MPs) and CNN to provide more features for LiDAR-DSM classification [16]. He et al. used spatial transformer networks (STN) to identify the best input of CNN for LiDAR classification [17]. Xia et al. used an ensemble classifier to process morphological features which fuses hyperspectral (HS) images and LiDAR-DSM [18]. Ge et al. proposed a new framework for fusion of hyperspectral image and LiDAR data based on the extinction profiles, local binary pattern (LBP), and kernel collaborative representation classification [19].

AlexNet [20] won the ImageNet [21] competition in 2012, making the convolutional neural network (CNN) become the focus again. It is a breakthrough achievement in the history of CNN development. Since then, convolutional neural networks have been widely used in various image processing fields [22,23,24,25,26,27,28,29,30,31,32]. Compared with the traditional machine learning algorithms, the three characteristics of CNN (receptive field, weights sharing, and subsampling) make it learn characteristics from the images directly. Researchers have proposed various CNNs with different structures and obtained good classification results [33,34,35,36]. These excellent CNNs are designed to achieve higher classification accuracy by designing deeper and more complex structures. However, they have relatively many structural parameters and may require large storage space and computing resources. 

SqueezeNet is a lightweight structure with a smaller number of structural parameters and less calculations whose structure and classification accuracy satisfy the application requirements as well [37]. SqueezeNet has only 1 × 1 and 3 × 3 convolution kernels and its purpose is not to obtain the best classification accuracy but to simplify the complexity of the network and achieve the classification accuracy similar to a public network. Octave Convolution (OctConv) is mainly used to deal with feature mapping of multiple spatial frequencies and reduce spatial redundancy. It is a single, generic, and plug-and-play convolution unit that can replace ordinary convolution directly and it does not need to adjust the network structure. In addition, the features of adjacent pixels in one image have similarities; however, the convolution kernel in traditional CNNs sweeps each location and stores its own feature description independently. It ignores spatial consistency, so that the feature maps have a large amount of redundancy in the spatial dimension. OctConv scales a portion of the feature maps and then communicates with the unscaled portion to reduce spatial redundancy and enhance feature utilization [38]. In this article, we propose a novel dual neural architecture, OctSqueezeNet, which combines SqueezeNet with OctConv, which improves LiDAR-DSM data classification accuracy with less structural parameter memory. 

In this article, OctSqueezeNet was applied to LiDAR data classification. The main contributions are written as follows:

SqueezeNet combined with OctConv was used to LiDAR data classification for the first time, which improved the classification accuracy and reduced the storage space occupied by the network. We replaced the original convolutional layer in OctConv with the Fire modules in SqueezeNet to compensate for the shortcomings of SqueezeNet, which had less extracted feature information due to lots of 1 × 1 convolution kernel.

## 2. SqueezeNet Design Architecture

**Figure 1:**
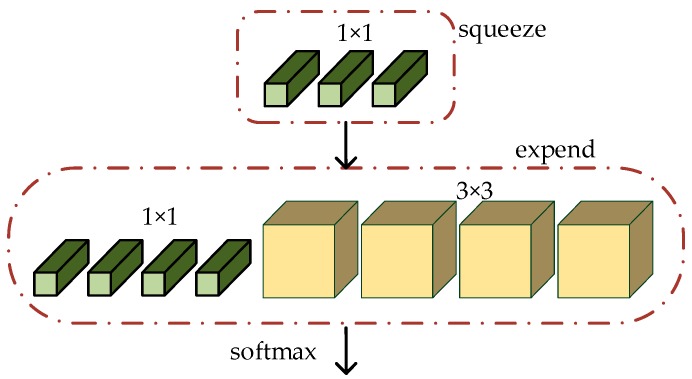
Structure of Fire module.

The main objective of SqueezeNet is maintaining competitive accuracy with few parameters. To achieve this goal, three main strategies were adopted. Firstly, the 3 × 3 filter was replaced by the 1 × 1 filter for which has fewer parameters. Secondly, we reduced the number of input channels to the 3 × 3 filters. Finally, we performed subsampled operations in the later stages of the network to make the convolution layer with large activation. SqueezeNet drew on the idea of the Inception module [23] to design a Fire module with a squeeze layer and an expand layer. The structure of Fire module is shown in Figure 1. In order to reduce the number of channels for input elements, the squeeze layer used a 1 × 1 convolution kernel to compress the input elements. The expansion layer used the 1 × 1 and 3 × 3 convolution kernels for multi-scale learning and concatenating.

**Figure 2:**
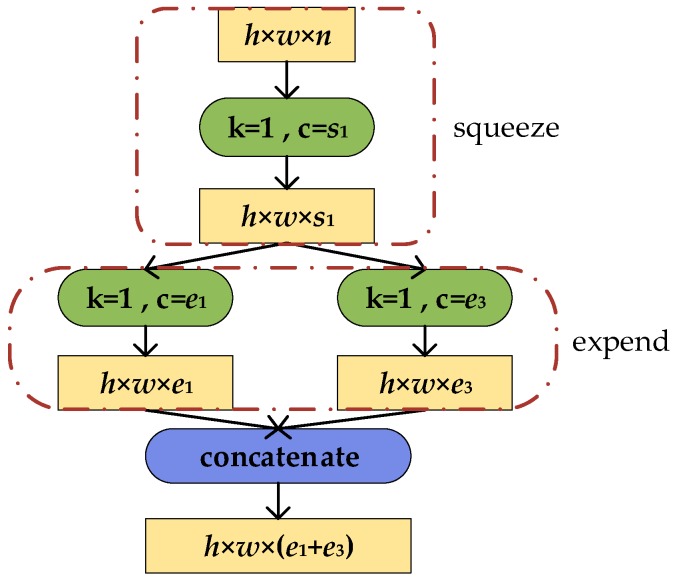
Operation process of Fire module.

The operation process of the Fire module is shown in Figure 2. The size of the input feature maps is *h* × *w* × *n*. Firstly, the input feature maps pass through the squeeze layer and obtain the output feature maps with a size of *h* × *w* × *s*_1_. The size of the feature maps is unchanged but the channels reduce from n to *s*_1_. The output feature maps of squeeze layer are sent into 1 × 1 and 3 × 3 convolution kernels in expand layer, respectively. Then concatenate the result of convolution. Finally, only the number of channels changes to *e*_1_ + *e*_3_. In order to enable the output activation of the 1 × 1 and 3 × 3 filters of the extension module to have the same height and width, the boundary zero filling operation with 1 pixel is performed for the input of the 3 × 3 filters in the extension module. Rectified Linear Unit (ReLU) is applied to the activation of squeeze layer and an expand layer. Meanwhile, there is no full-connection layer in SqueezeNet.

## 3. Octave Convolution

Natural images can be decomposed into low spatial frequency components and high spatial frequency components. Similarly, the feature maps of the convolutional layers can also be decomposed into feature components of different spatial frequencies. The low frequency components are used to describe the structure with the smooth changes, and the high frequency components are used to describe the fine details of the fast changes. 

**Figure 3:**
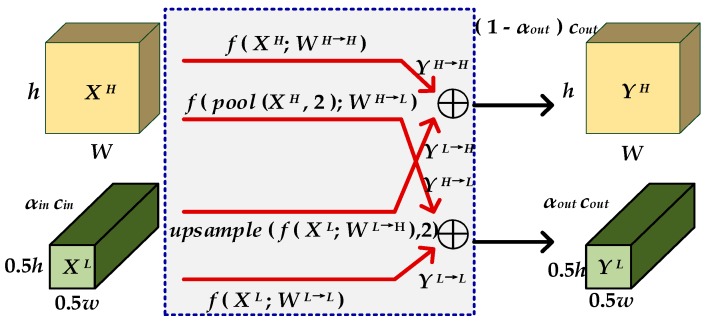
Structure of OctConv.

As shown in Figure 3, the components of high and low frequency of the image are written as *X^H^* and *X^L^*, and their corresponding outputs are *Y^H^* and *Y^L^* after the convolution operation, where α ∈ (0,1) represents the ratio of the low frequency channels. The arrows above and below represent that the feature maps with the same frequency self-update their information by convolution operation, and the crossed arrows help to exchange information between the two frequencies by pooling, upsample, and add operations. In the convolution operation, *W^H^* responses are both from *X^H^* to *Y^H^* and from *X^L^* to *Y^H^*, that is *W^H^* = [*W^H→H^*,*W^L→H^*]. *W^H→H^* is traditional convolution, and the size of input and output images are the same. *W^L^*^→*H*^ upsamples the input image first and then performs traditional convolution, while *W^H^*^→*L*^ pools the input image first. As shown as Equations (1) and (2), the high and low frequency feature maps are stored in different groups. Sharing information between adjacent locations can reduce the spatial resolution of the low-frequency group and spatial redundancy.

(1) $$Y^{H}=Y^{H\to H}+Y^{L\to H}$$

(2) $$Y^{L}=Y^{L\to L}+Y^{H\to L}$$

The specific calculation process is written as Equations (3) and (4).

(3) $$Y^{H}=f\left(X^{H};W^{H\to H}\right)+upsample\;\left(f\left(X^{L};W^{L\to H}\right),2\right)$$

(4) $$Y^{L}=f\left(X^{L};W^{L\to L}\right)+f\left(pool\left(X^{H},2\right);W^{H\to L}\right)$$

## 4. OctSqueezeNet for LiDAR Classification

**Figure 4:**
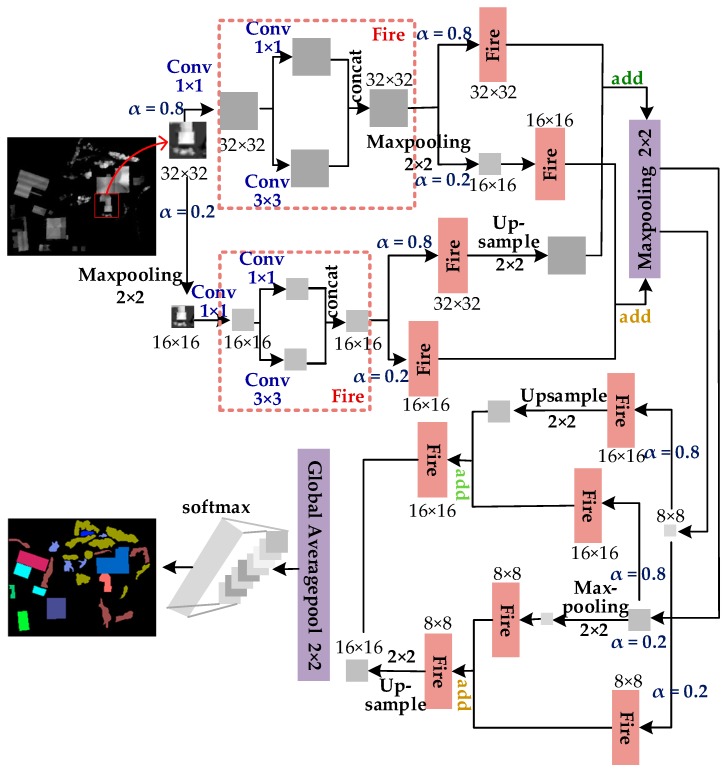
Architecture of OctSqueezeNet.

As shown in Figure 4, we rescaled the feature maps with different resolutions to the same spatial resolution and concatenated their feature channels, forming a dual neural architecture similar to multi-layers called OctSqueezeNet [39] for LiDAR data classification processing.

First, random selection of 20% from the input feature map performed a 2 × 2 maxpooling operation to halve the size to 16 × 16, and the remaining feature maps maintained the original size, 32 × 32. The two parts were sent separately to different Fire modules to obtain their respective output feature maps. The size of feature maps did not change through the Fire module. We repeated the above operation for the 32 × 32 output feature maps. At the same time, 80% of the 16 × 16 output feature maps were sent to a Fire module and a 2 × 2 upsampling operation was performed on the corresponding output to restore the size to 32 × 32; the remaining 20% of these were sent to another Fire module. Feature maps with the same size in different sections were combined to form the dual outputs, then sent them to the average pooling layer to obtain output feature maps with size 16 × 16 and 8 × 8. 

As shown in the lower part of Figure 4, the above operation was repeated for these 16 × 16 output feature maps. Meanwhile, 80% of the these 8 × 8 output feature maps were only sent to a Fire module; the remaining 20% were sent to a Fire module and then the outputs the outputs after this Fire module were sent to a 2 × 2 upsampled layer to restore to the size of 16 × 16.

The feature maps with the same size in different parts were combined to obtain 8 × 8 and 16 × 16 and, respectively, sent to the Fire module, shown as the lower part of Figure 4. Then we upsampled the 8 × 8 feature maps to 16 × 16. The 16 × 16 feature maps were merged to obtain a single output and downsampled to 8 × 8. Finally, softmax was used for data classification.

### 4.1. Adaptive Learning Optimization Algorithm

Adam optimization algorithm is an extension of the stochastic gradient descent (SGD) algorithm. It is a step-optimization algorithm based on the gradient stochastic objective function and low-order moment adaptive estimation based on training data. It can replace the SGD and update the weight of the neural network iteratively based on the training data. The method estimates the different parameters by the first order matrix, *m_t_*, and the second order matrix, *n_t_*, of the gradient to calculate the adaptive learning rate. The process is shown as Equations (5)–(7):

(5) $$m_{t}=\mu \times m_{t-1}+\left(1-\mu \right)\times g_{t}$$

(6) $$n_{t}=V\times n_{t-1}+\left(1-V\right)\times g_{t}^{2}$$

(7) $$m_{t}^{\prime }=\frac{m_{t}}{1-\mu^{t}}$$

(8) $$n_{t}^{\prime }=\frac{n_{t}}{1-v^{t}}$$

The *m_t_*′ and *n_t_*′ are the correction for *m_t_* and *n_t_*, which approximate the expected unbiased estimate, have no additional requirements for memory, and can be adjusted dynamically according to the gradient, where -$m_{t}^{\prime }/\sqrt{n_{t}^{\prime }}+\varepsilon$ forms a dynamic constraint on the learning rate and it has a clear scope.

### 4.2. Loss and Activate Function

The activate function of the structure in this article is ReLU. As shown as Equation (9), it causes a part of the output of neuron to zero, which results in sparseness of the network and reduces the interdependence of parameters, alleviating the problem of overfitting. The calculation of the whole process saves a lot of time.

(9) g(x)=max(0,x)

Because our dataset is a multi-category dataset, softmax is adopted as the final classifier of the network model. Shown in Equation (10), softmax is used as the exponential operation, which can increase the comparison of the large values and small values to improve learning efficiency.

(10) $$a_{j}^{L}=\frac{e^{Z_{j}^{L}}}{\sum_{K}e^{Z_{K}^{L}}}$$

where $Z_{j}^{L}$ represents the input of the *jth* neuron of the *Lth* layer (usually the last layer), $a_{j}^{L}$ represents the output of the *jth* neuron in the *Lth* layer, and *e* represents the natural constant. $\sum_{K}e^{Z_{K}^{L}}$ represents the sum of the inputs of all neurons in the *Lth* layer. Therefore, as Equation (11), the corresponding loss function is a combination of softmax and cross-entropy loss.

(11) $$Loss_{i}=-\log y_{i}=-\log\frac{e^{Z_{j}^{L}}}{\sum_{K}e^{Z_{K}^{L}}}$$

## 5. Experimental Results and Analysis

### 5.1. Datasets Description

**Figure 5:**
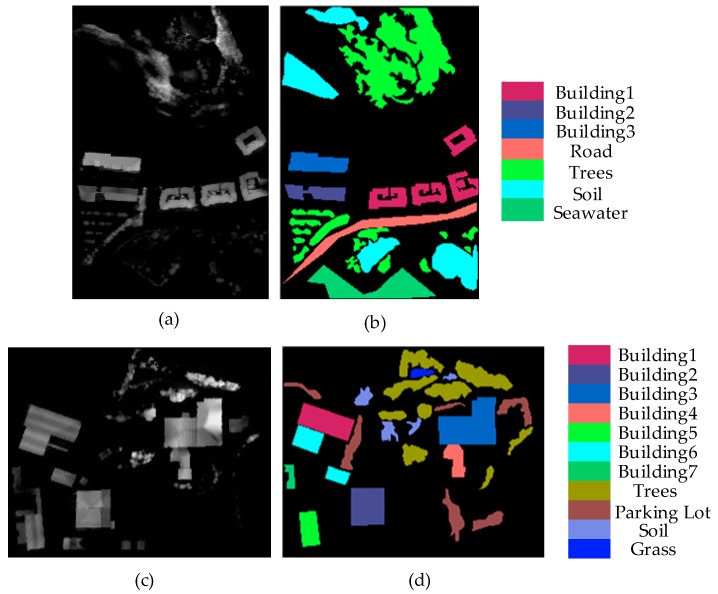
Bayview Park (up) and Recology (down) datasets: (**a**) Digital surface models (DSM) of Bayview Park, (**b**) groundtruth of Bayview Park, (**c**) DSM of Recology, (**d**) groundtruth of Recology.

This article conducted experiments on two different datasets, Bayview Park and Recology, to evaluate the performance of the proposed classification method. They are the public datasets of the 2012 IEEE International Remote Sensing Image Convergence Competition and collected in the city of San Francisco, CA, USA. The Bayview Park dataset has a size of 300 × 200 pixels, the spatial resolution of 1.8 meters and marks 7 land classes. The Recology dataset consists of 200 × 250 pixels with a spatial resolution of 1.8 meters and contains 11 land classes. Figure 5 shows the DSM maps and the groundtruth maps, respectively, for Bayview Park and Recology datasets.

### 5.2. Experimental Set-Up

Experiments used the TensorFlow under windows as the backend, encoded with Keras and Python. We divided the datasets into two parts: Training samples and test samples. The number of training samples (i.e., 400, 500, 600, and 700) was selected randomly and the rest were used as test sets. And we adopted overall accuracy (OA), average accuracy (AA), and Kappa as objective evaluation criteria. In the experiments, SqueezeNet only used convolutions size with 1 × 1 and 3 × 3, the input features of both datasets were 32 × 32 pixels. DSM data were linearly mapped to (−1 1) and the gradient optimization algorithm selected Adam. The kernel function of SVM was set to radial basis function (rbf) and the coefficient of rbf was default to auto. The penalty parameter of the error terms was 100. The initial learning rate for Bayview Park and Recology datasets both were 0.001 in CNN, and they were set to 0.0005 and 0.001, respectively, in OctConv. In SqueezeNet and OctSqueezeNet, the initial learning rates for two datasets were both set to 0.0005. For the two datasets, the ratio of pooling for feature maps in the first input layer and middle layers were all set to 0.2. The last output layer did not perform pooling. In this article, the initial learning rate for Bayview Park and Recology datasets both were 0.001 in CNN, and they were set to 0.0005 and 0.001, respectively, in OctConv; in SqueezeNet and OctSqueezeNet, the initial learning rates for the two datasets were both set to 0.0005.

#### 5.2.1. Bayview Park Dataset

**Figure 6:**
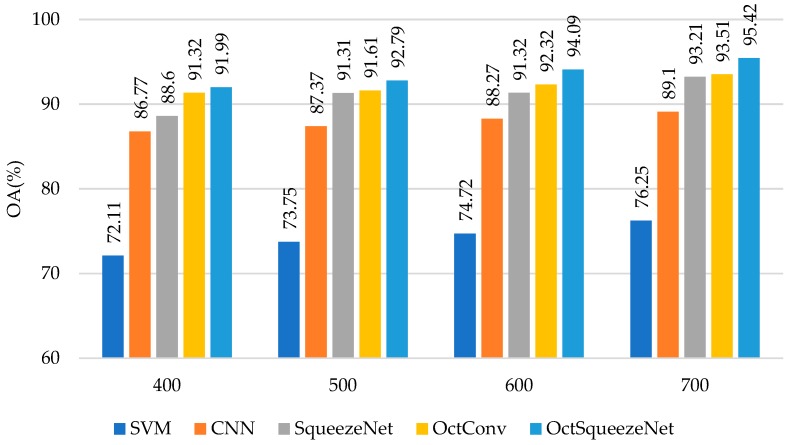
The classification results of different methods on Bayview Park.

The experiments ran on a 3.2-GHz CPU with a GTX 1060 GPU card. As shown in Table 1, OctSqueezeNet achieved the best results on OA, AA, and Kappa when different numbers of training samples were selected. When 700 samples were selected, the best OA was 95.42%, increasing 1.91%, 2.21%, 6.32%, and 19.17% compared to OctConv, SqueezeNet, CNN, and SVM, respectively. Figure 6 uses the bar chart to show the accuracy of each comparison method when different numbers of samples were selected. It can be seen intuitively that the accuracy of OctSqueezeNet proposed by us was always higher than that of other methods.

**Figure 7:**
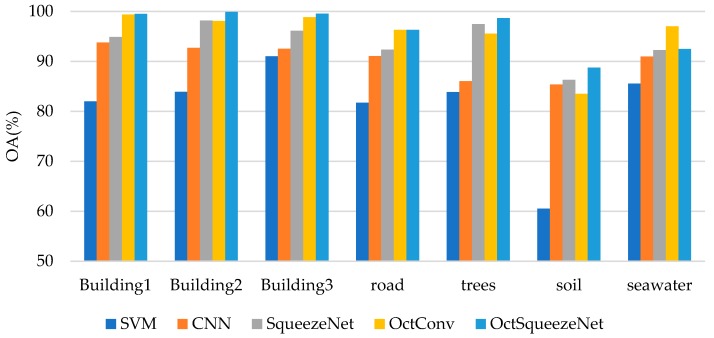
Classification results of different methods for each class on Bayview Park.

**Figure 8:**
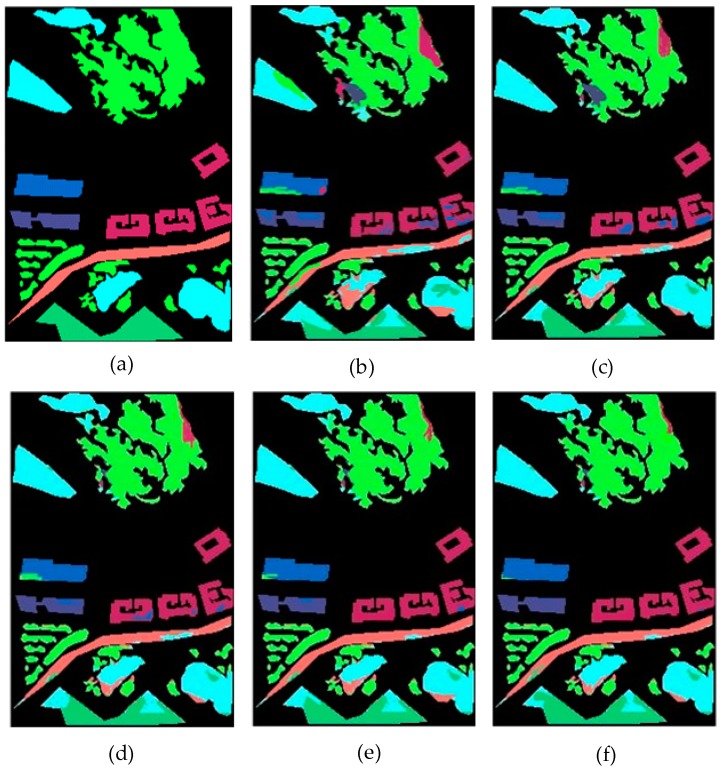
Classification results on Bayview Park: (**a**) Groundtruth map, (**b**) support vector machine (SVM), (**c**) convolutional neural network (CNN), (**d**) SqueezeNet, (**e**) Octave Convolution (OctConv), and (**f**) OctSqueezeNet.

Table 2 and Figure 7 show the classification accuracy of each class. OctSqueezeNet had a good effect on classification of different land classes. Figure 8 shows the classification results of different networks for Bayview Park visually through the false color maps. It can be seen from the classification results, as the precision increased, the mis-division area of the feature gradually decreased.

#### 5.2.2. Recology Dataset

**Figure 9:**
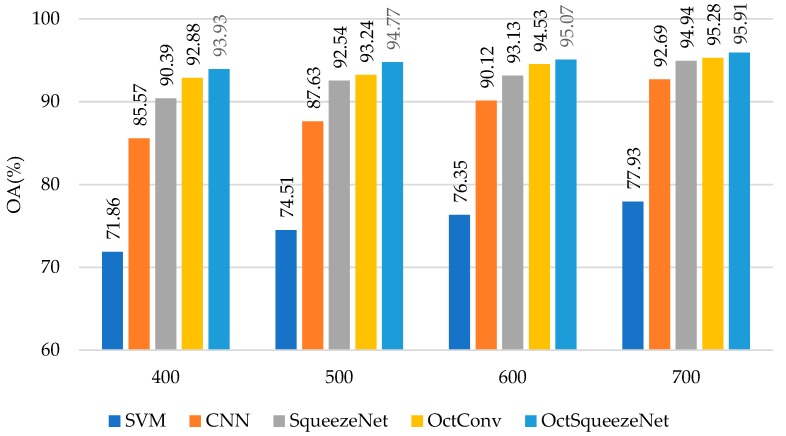
The classification results of different methods on Recology.

As shown in Table 3, OctSqueezeNet also achieved the best results for Recology dataset on OA, AA, and Kappa when selecting different numbers of training samples. When selecting 700 samples, the best OA was 95.91%, increasing 0.63%, 0.97%, 3.22%, and 17.98% compared to OctConv, SqueezeNet, CNN, and SVM, respectively. Figure 9 shows that the accuracy of the five methods grew steadily with the number of samples increasing. The accuracy of OctSqueezeNet was always the highest.

**Figure 10:**
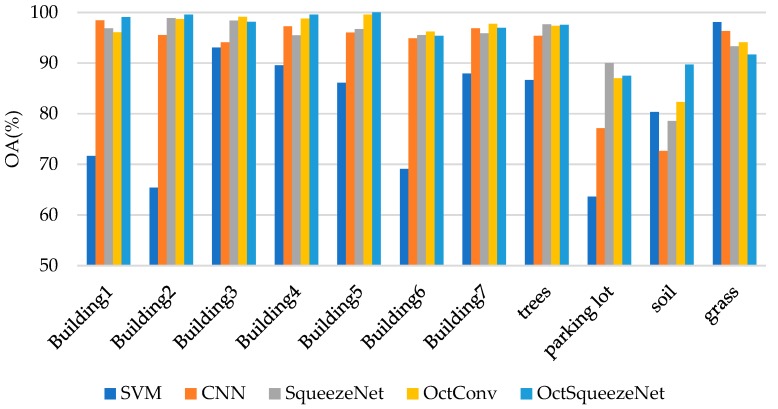
Classification results of different methods for each class on Recology.

**Figure 11:**
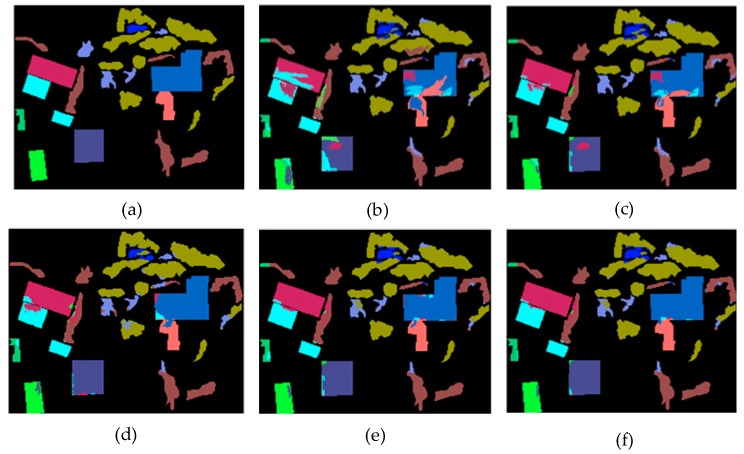
Classification results on Recology: (**a**) Groundtruth map, (**b**) SVM, (**c**) CNN, (**d**) SqueezeNet, (**e**) OctConv, and (**f**) OctSqueezeNet.

Table 4 and Figure 10 also prove that OctSqueezeNet had better classification effects on different classes. Figure 11 shows the classification results of the land classes and the area of the wrong division was also less and less with the increase of precision.

### 5.3. Selection of Experimental Parameters

The number of parameters and the model size of the proposed method in this article were compared with the classical methods. In order to adapt to the Bayview Park and Recology datasets, we made some adjustments to the structure of these classic algorithms. Next, the adjustments made in each architecture are described.

(1) SqueezeNet: The first change was the addition of batch normalization, which was not present in the original architecture. Then dropout was not used in the last convolution layer. Finally, we changed the size of the kernel on the first convolution layer from original 7 × 7 to 3 × 3.
(2) AlexNet: Its original architecture contained eight weights layers; the first five layers were convolution and the rest of the layers were fully connected. In comparison experiments, the first convolution layer changed the stride from 4 to 1. The output of the last fully connected layer was fed into a 7-way softmax for the Bayview Park dataset and an 11-way softmax for the Recology dataset.
(3) ResNet-34: Original Resnet-34 had 4 sections which were composed of 3, 4, 6, and 3 identity blocks, respectively. The number of filters was 64, 128, 256, and 512, respectively, in each identity block of the four sections. In our experiments, the kernel of the first convolution layer was changed from 7 to 3. The number of filters was modified to 16, 28, 40, and 52, respectively. The output of the last fully connected layer was set the same as AlexNet.

As shown in Table 5, compared with some classical CNN algorithms, OctSqueezeNet proposed by us had higher precision, and reduced the number of parameters and model size significantly. When 700 training sets were selected, the OA of OctSqueezeNet on the Bayview Park dataset reached 95.42%, increasing 2.33%, 1.67%, and 2.21% compared with AlexNet, ResNet-34, and SqueezeNet, respectively; but the number of parameters of OcSqueezeNet was only 0.32M, which is 29.67M, 0.1M, and 0.92M less than the number of parameters of AlexNet, ResNet-34, and SqueezeNet, respectively. The model size was only 1.38M, which is 113M, 0.45M, and 3.45M less than the model size of AlexNet, ResNet-34, and SqueezeNet, respectively. The best OA of OcSqueezeNet on the Recology dataset was 95.91% and it was 1.64%, 0.86%, and 0.97% higher than AlexNet, ResNet-34, and SqueezeNet. Its number of parameters was the least (0.33M) compared with the other three methods. The number of parameters of AlexNet, ResNet-34, and SqueezeNet was 114.51M, 1.84M, and 4.85M, respectively. The model size of OctSqueezeNet was also the smallest (1.37M) of these four methods, which decreased 113.14M, 0.47M, and 3.48M compare to the model size of AlexNet, ResNet-34, and SqueezeNet.

Additionally, the training and test time are shown in Table 5 where 700 samples were selected as training sets and experiments ran 150 epochs. For Bayview Park data set, the training time of AlexNet, Resnet-34, SqueezeNet and OctSqueezeNet are 212.13 s, 116.35 s, 75.82 s and 108.82 s respectively. The test time of them are 223.12 s, 160.17 s, 65.43 s and 151.07 s respectively. For Recology data set, the training time of the four methods are 222.94 s, 169.94 s, 38.04 s and 119.62 s respectively. The test time of them are 230.52 s, 207.63 s, 44.12 s and 191.54 s respectively. The time of OctSqueezeNet was longer than SqueezeNet. Although the parameters were reduced, the structural branches were more complicated, which affected the transfer time. However, though the time was slower than SqueezeNet itself, it was acceptable compared to the greatly improved accuracy and the reduced number of parameters and model size. OctSqueezeNet was much faster than AlexNet and Resnet-34.

## 6. Conclusions

This article designed a dual neural architecture OctSqueezeNet to classify LiDAR-DSM data. Using SqueezeNet alone without combining with OctConv, the network parameters were more and the model size was larger. Most importantly, the classification accuracy of the dataset was reduced significantly. Because SqueezeNet replaced the traditional 3 × 3 convolution kernels with a large number of 1 × 1 convolution kernels, which lost more extracted features relatively. The OctConv did not change the size of the convolution kernel. By reducing the size of a part of the feature maps, it was equivalent to expanding the receptive field to obtain more global feature information. The feature maps with the original size were equivalent to extracting more detailed feature information, because the receptive field had no change. This can compensate for the loss of SqueezeNet in feature extraction. Because there were only 1 × 1 and 3 × 3 convolution kernels, it also made parameters of a network fewer and the model size smaller.

Overall, combining SqueezeNet with OctConv gave better classification precision of datasets and less space for the storage model. The experiment results indicate that OctSqueezeNet achieved 95.42% and 95.66%, respectively, in terms of OA on the Bayview Park and Recology datasets when the number of training samples was 700, which were better than other classification methods. At the same time, for the two datasets, the number of parameters of OctSqueezeNet achieved 0.32M and 0.33M, respectively, and the model size of OctSqueezeNet achieved 1.38M and 1.37M, respectively. They were lower than some other methods. The combination of SqueezeNet and OctConv opens a new window for LiDAR data classification by fully extracting its spatial information.

## Figures and Tables

**Table 1:** Classification results on Bayview Park dataset.

Training of Different Samples	Index	SVM	CNN	SqueezeNet	OctConv	OctSqueezeNet
400	OA%	72.11 ± 2.02	86.77 ± 2.11	88.60 ± 1.56	91.32 ± 0.41	91.99 ± 0.81
	AA%	77.08 ± 1.43	88.01 ± 1.32	89.04 ± 0.47	93.02 ± 0.32	93.21 ± 0.43
	K×100	63.11 ± 1.89	82.92 ± 1.77	85.42 ± 2.01	88.30 ± 0.66	89.48 ± 1.00
500	OA%	73.75 ± 2.40	87.37 ± 1.07	91.31 ± 2.81	91.61 ± 0.63	92.79 ± 0.41
	AA%	78.45 ± 2.07	89.21 ± 2.99	90.59 ± 2.05	93.37 ± 1.21	95.02 ± 0.90
	K×100	65.41 ± 0.84	84.62 ± 1.52	88.44 ± 1.65	88.47 ± 1.43	90.48 ± 0.47
600	OA%	74.72 ± 2.03	88.27 ± 0.71	91.32 ± 1.03	92.32 ± 0.49	94.09 ± 1.23
	AA%	78.87 ± 1.10	89.44 ± 2.64	92.11 ± 1.57	94.30 ± 0.51	95.75 ± 1.25
	K×100	66.74 ± 1.88	85.88 ± 2.01	89.63 ± 1.43	89.89 ± 0.72	92.23 ± 1.64
700	OA%	76.25 ± 0.74	89.10 ± 2.09	93.21 ± 0.63	93.51 ± 0.77	95.42 ± 0.91
	AA%	81.21 ± 2.25	90.11 ± 0.88	93.33 ± 0.79	95.64 ± 1.56	96.43 ± 1.37
	K×100	68.70 ± 2.14	86.52 ± 2.70	91.11 ± 2.14	91.38 ± 1.04	93.99 ± 1.97

**Table 2:** Classification results of each class on Bayview Park dataset.

NO.	Classes	SVM	CNN	SqueezeNet	OctConv	OctSqueezeNet
1	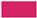	82.00 ± 4.21	93.78 ± 1.76	94.89 ± 1.33	99.40 ± 0.41	99.52 ± 0.09
2	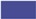	83.88 ± 3.32	92.71 ± 1.04	98.16 ± 1.84	98.08 ± 1.53	99.93 ± 0.07
3	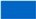	91.01 ± 5.20	92.53 ± 1.82	96.12 ± 1.14	98.84 ± 1.36	99.54 ± 0.46
4	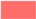	81.70 ± 4.31	91.05 ± 1.24	92.37 ± 0.33	96.29 ± 0.88	96.29 ± 2.77
5	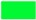	83.85 ± 1.99	86.03 ± 1.81	97.45 ± 3.11	95.54 ± 0.67	98.67 ± 0.88
6	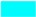	60.53 ± 3.39	85.37 ± 1.79	86.29 ± 3.02	83.51 ± 0.53	88.75 ± 3.26
7	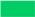	85.53 ± 2.74	90.98 ± 2.64	92.25 ± 1.98	97.01 ± 2.51	92.47 ± 2.30

**Table 3:** Classification results on Recology dataset.

Training of Different Samples	Index	SVM	CNN	SqueezeNet	OctConv	OctSqueezeNet
400	OA%	71.86 ± 1.05	85.57 ± 1.37	90.39 ± 0.65	92.88 ± 1.07	93.93 ± 0.61
	AA%	72.69 ± 2.43	88.97 ± 2.13	89.02 ± 0.18	91.17 ± 1.56	93.63 ± 0.17
	K×100	66.62 ± 0.88	82.35 ± 1.71	88.67 ± 3.21	91.28 ± 0.73	92.79 ± 0.74
500	OA%	74.51 ± 0.77	87.63 ± 1.43	92.54 ± 2.01	93.24 ± 1.14	94.77 ± 0.83
	AA%	77.64 ± 3.26	91.06 ± 0.39	91.28 ± 1.21	91.41 ± 0.57	93.72 ± 0.60
	K×100	69.73 ± 1.75	85.98 ± 0.29	87.30 ± 1.43	92.06 ± 1.20	93.79 ± 0.99
600	OA%	76.35 ± 2.33	90.12 ± 0.52	93.13 ± 0.71	94.53 ± 1.24	95.07 ± 0.48
	AA%	79.51 ± 1.31	88.77 ± 1.22	92.38 ± 1.72	94.02 ± 0.62	95.36 ± 1.15
	K×100	71.85 ± 2.01	86.89 ± 0.72	91.94 ± 2.13	93.51 ± 1.44	94.13 ± 0.63
700	OA%	77.93 ± 0.93	92.69 ± 1.69	94.94 ± 2.63	95.28 ± 1.38	95.91 ± 0.73
	AA%	81.00 ± 1.32	92.22 ± 1.47	94.29 ± 0.79	94.82 ± 1.16	95.89 ± 0.17
	K×100	73.70 ± 1.01	89.53 ± 2.18	93.19 ± 2.14	94.40 ± 1.09	95.13 ± 0.11

**Table 4:** Classification results of each class on Recology dataset.

NO.	Classes	SVM	CNN	SqueezeNet	OctConv	OctSqueezeNet
1	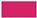	71.66 ± 0.94	98.42 ± 1.09	96.87 ± 1.20	96.05 ± 1.01	99.06 ± 0.94
2	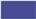	65.04 ± 0.74	95.53 ± 1.21	98.89 ± 1.10	98.67 ± 1.69	99.56 ± 0.44
3	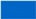	93.04 ± 1.04	94.09 ± 1.12	98.38 ± 1.99	99.11 ± 0.54	98.12 ± 1.43
4	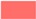	89.54 ± 2.33	97.25 ± 1.13	95.48 ± 1.25	98.80 ± 0.58	99.55 ± 0.45
5	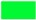	86.08 ± 1.20	96.01 ± 1.95	96.72 ± 2.16	99.59 ± 0.41	100
6	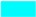	69.11 ± 1.12	94.87 ± 1.41	95.54 ± 1.42	96.21 ± 0.11	95.35 ± 1.81
7	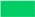	87.94 ± 3.11	96.83 ± 1.79	95.87 ± 2.01	97.74 ± 2.46	96.94 ± 1.64
8	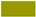	86.66 ± 1.11	95.36 ± 0.61	97.64 ± 0.61	97.36 ± 0.34	97.54 ± 2.01
9	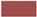	63.61 ± 2.12	77.14 ± 0.79	89.99 ± 1.64	86.99 ± 1.25	87.48 ± 1.27
10	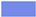	80.31 ± 4.34	72.62 ±1.46	78.53 ± 2.33	82.28 ± 2.12	89.68 ± 0.53
11	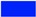	98.09 ± 1.87	96.31 ± 1.27	93.31 ± 0.48	94.09 ± 1.26	91.68 ± 1.61

**Table 5:** Performance comparison of 700 samples for different networks.

Data	Method	Params (Million)	OA (%)	Model Size (M)	Training Time (s)	Test Time (s)
Bayview Park	AlexNet	29.99	93.09 ± 0.66	114.38	212.13	223.12
	ResNet-34	0.42	93.75 ± 1.29	1.83	125.15	160.17
	SqueezeNet	1.24	93.21 ± 0.63	4.83	75.82	65.43
	OctSqueezeNet	0.32	95.42 ± 0.91	1.38	108.82	151.07
Recology	AlexNet	29.99	94.27 ± 0.74	114.51	222.94	230.52
	ResNet-34	0.41	95.05 ± 0.31	1.84	169.94	207.63
	SqueezeNet	1.25	94.94 ± 2.63	4.85	38.04	44.12
	OctSqueezeNet	0.33	95.91 ± 0.73	1.37	119.62	191.54
